# Infection of Semen-Producing Organs by SIV during the Acute and Chronic Stages of the Disease

**DOI:** 10.1371/journal.pone.0001792

**Published:** 2008-03-12

**Authors:** Anna Le Tortorec, Roger Le Grand, Hélène Denis, Anne-Pascale Satie, Karim Mannioui, Pierre Roques, Anne Maillard, Sylvanne Daniels, Bernard Jégou, Nathalie Dejucq-Rainsford

**Affiliations:** 1 INSERM U625, Rennes, University of Rennes I, Groupe d'Etude de la Reproduction chez l'Homme et les Mammifères, IFR 140, Campus de Beaulieu, Rennes, France; 2 CEA, Service d'immuno-virologie, DSV/iMETI, Fontenay-aux-Roses, France; 3 Unité de Rétrovirologie, Centre Hospitalier Universitaire Régional Pontchaillou, Rennes, France; Institut Pasteur Korea, Republic of Korea

## Abstract

**Background:**

Although indirect evidence suggests the male genital tract as a possible source of persistent HIV shedding in semen during antiretroviral therapy, this phenomenon is poorly understood due to the difficulty of sampling semen-producing organs in HIV+ asymptomatic individuals.

**Methodology/Principal Findings:**

Using a range of molecular and cell biological techniques, this study investigates SIV infection within reproductive organs of macaques during the acute and chronic stages of the disease. We demonstrate for the first time the presence of SIV in the testes, epididymides, prostate and seminal vesicles as early as 14 days post-inoculation. This infection persists throughout the chronic stage and positively correlates with blood viremia. The prostate and seminal vesicles appear to be the most efficiently infected reproductive organs, followed by the epididymides and testes. Within the male genital tract, mostly T lymphocytes and a small number of germ cells harbour SIV antigens and RNA. In contrast to the other organs studied, the testis does not display an immune response to the infection. Testosteronemia is transiently increased during the early phase of the infection but spermatogenesis remains unaffected.

**Conclusions/Significance:**

The present study reveals that SIV infection of the macaque male genital tract is an early event and that semen-producing organs display differential infection levels and immune responses. These results help elucidate the origin of HIV in semen and constitute an essential base to improving the design of antiretroviral therapies to eradicate virus from semen.

## Introduction

While semen represents the main vector of human immunodeficiency virus type 1 (HIV-1) dissemination worldwide, the origin of the free HIV-1 particles and infected seminal leukocytes contaminating this bodily fluid is poorly understood. A number of studies have shown that semen represents a viral compartment distinct from the blood [1]–[13], suggesting that local sources within the male genital tract (MGT) contribute virus particles and infected cells to semen.

Semen is composed of secretions and cells from the testes, epididymides, prostate, seminal vesicles and urethral glands. To shed light onto the origin of HIV in semen, a necessary prerequisite is to determine whether and which semen-producing organs are productively infected by the virus and susceptible to seed virus or infected cells into semen. Importantly, despite antiretroviral therapy achieving an undetectable blood viral load, virus release can persist in semen [3], [14]–[18], leading to an increase in sexual transmission of drug resistant strains [19]–[21]. Therefore, identification of the different HIV sources within the MGT is critical for more efficient control of HIV transmission.

While HIV-1 and simian immunodeficiency virus (SIV) were found in a number of male genital organs from AIDS deceased men [22]–[24] and macaques [25], very little is known about HIV/SIV infection of the male reproductive tract during the asymptomatic phase of the infection. However, model estimates suggest HIV transmission rates to be highest during the acute stage of the infection, when semen is the most infectious [26], [27], and lowest during the chronic phase [28]–[31]. Whether this reflects differential levels of contamination of the male genital organs during the course of the infection has never before been investigated.

There are a number of practical and ethical reasons which prevent the design of time-course studies of HIV infection of the MGT in men. To verify the hypothesis that various male genital tract organs are infected early and could thus represent a primary source of virus shed in the semen, it is therefore necessary to use an appropriate animal model. Since the experimental infection of macaques by SIV represents the best animal model in which to study HIV infection *in vivo* [32], the testes, epididymides, and accessory glands (prostate and seminal vesicles) of SIV-infected macaques in the acute and chronic stages of the disease were examined during this study. Using molecular- and cell biological techniques, we established the kinetics of infection within the MGT and correlation with blood viremia. Furthermore, we identified viral target tissues and cells and demonstrated that all infected organs except testis showed an inflammatory response.

## Materials and Methods

### Animals and infection

Fourteen adult male cynomolgus macaques (*Macaca fascicularis*) (3–4 years old, body weight>5 Kg, all mature as attested by the presence of full spermatogenesis) imported from Mauritius were included in the present study, having been previously screened for pathogens [33]. Animals were housed at the primate facilities of CEA, France and handled in accordance with EC guidelines (*Journal Officiel des Communautés Européennes*, L358, December 18, 1986). Eleven macaques were intravenously inoculated with 50 AID_50_ (50% animal infectious dose) of pathogenic cell-free SIVmac251 in 1 ml of phosphate-buffered saline (PBS). The generation and titration of the SIVmac251 virus stock have been described elsewhere [33].

### Specimen collection and blood viral load measurement

Blood was periodically collected throughout the infection and at the time of euthanasia. Plasma viral loads (PVLs) and peripheral CD4 cell counts were assessed as previously described [34]. Tissues were collected immediately after euthanasia and exsanguinations of the animals, extensively washed and cut into fragments weighing about 300 mg each. The fragments were either stored at −80°C or fixed in 4% formaldehyde.

### Nucleic acids extraction

Total RNA and DNA were extracted from 2 distinct fragments of each tissue using the RNeasy isolation maxi kit or the QIAamp DNA maxi kit (both Qiagen, Courtaboeuf, France), respectively. RNA samples were depleted of contaminating DNA by DNase treatment (Promega, Charbonnières, France) and submitted to RT reactions, using random hexamer primers (Boehringer-Mannheim, Mannheim, Germany) and M-MLV-Reverse Transcriptase (Invitrogen, Cergy-Pontoise, France). Total DNA from PBMC were isolated using a commercial kit (Genomic DNA from tissue, Macherey-Nagel, GmbH & KG, Germany).

### Nested PCR

A previously described, sensitive nested PCR [35] was used to detect SIV DNA. In order to increase the chances of detection of focal infection of the genital tissues, 2 independent fragments of each tissue were assayed in a minimum of 54 PCR reactions, each performed on 500 ng of extracted DNA. The sensitivity was 100% for a detection threshold of 10 copies of SIVmac251 gag DNA plasmid in 500 ng of exogenous DNA, and 33% for a detection threshold of 1 copy. Results were expressed as percentages of SIV gag positives PCR.

### Viral DNA quantification

DNA extracted from 2 independent fragments was analyzed in duplicate in real time PCR Taqman assay using the Platinium qPCR SuperMix UDG kit (Invitrogen) and previously described SIV gag primers and probe [34]. The reaction, data acquisition and analysis were performed with the ABI PRISM 7000 Sequence detection System (Applied Biosystems, Foster City, CA, USA). SIV DNA copy number in unknown samples was inferred by plotting the threshold cycle (Ct) value against a calibration curve (gag SIVmac251 DNA plasmid, linear dynamic range 10 to 10^7^ copies). Genomic normalizing GAPDH gene was simultaneously amplified, using previously published primer set and probe [36]. Results were expressed as SIV DNA copy numbers per copy of GAPDH.

### Cloning and phylogenetic analysis

Genital tissues DNA and PBMC DNA as well as blood plasma cDNA were submitted to nested PCR to amplify a 590-bp fragment encompassing the V1-V2 region of the SIV envelope gene, as previously described [37]. To reduce the possibility that differences between MGT, blood and serum sequences were caused by sampling errors, we performed the extraction, amplification and direct sequencing of the PCR products at least twice. Clones were sequenced from each of the two or three extractions as described below. PCR products were inserted into a plasmid with the TOPO 4TA cloning kit (Invitrogen). Ecoli Top10 (Invitrogen) were transformed and a minimum of 10 colonies were selected by PCR using inner primers. The inserts from purified plasmid were sequenced using an automated sequencer (Qiagen). Sequences accession numbers are AM397301 to AM397432. V1-V2 sequences were then submitted to phylogenetic analysis. After hand correction of crude sequences in MEGA3, alignments were performed using the reference sequences from SIVmac239 as root (Ac# M33262). Multiple sequences were aligned using CLUSTALW (1.8) [38] and adjusted using the alignment editor Se-Al (version 2.0; available from http://evolve.zoo.ox.ac.uk). Ambiguous regions and all sites including a gap were removed from the alignment. Phylogenetic trees were built using PAUP* version 4b10 [39] using a sequence evolution model chosen with Modeltest v3.06 [40]. The reliability of the branching order was estimated by performing bootstrap analysis (100 replicates). Only significant values above 50% were indicated on the branches.

### Immunohistochemistry

The following human mAbs and matching isotype controls were used at the indicated concentrations: anti-HLA-DR (TAL.1B5, 0,6 µg/ml), anti-CD68 (KP1, 1.2 µg/ml), anti-CD3 (F7.2.38, 6.75 µg/ml) (all from DAKO), anti-CD4 (1F6, Novocastra, 2.5 µg/ml), anti-TIA-1 (2G9, Immunotech, 1 µg/ml), anti-Pen5 (5H10 clone [41], neat) with mouse IgG1 isotype control (DAKO); anti-SIV gag p27 (ARP 397, CFAR, 1/100), anti-CD20 (L26, DAKO, Trappes, France, 0.44 µg/ml), anti-CCR5 (2D7, BD Biosciences, 25 µg/ml), anti-CXCR4 (12G5, NIBSC, 25 µg/ml) with mouse IgG2a control (BD Biosciences). A rabbit polyclonal Ab to DEAD-box protein 4 (DDX4, Abcam, 2 µg/ml) was used with rabbit IgG isotype control (Jackson Immunoresearch Laboratories, West Grove, PA, USA) to specifically stain spermatogenic cells [42]. Immunohistochemistry was performed as previously described [43]. No staining was ever observed with isotope control antibodies or control serum. A minimum of three sections from distinct areas were observed per animal. For quantitative and semi-quantitative measurement, cell counts were performed using the Cast software (Olympus) on two sections from three animals per group. In uninfected animals, stained positive cells were counted in 100 randomly selected fields/section at a magnification of 40×. For CD3+T cell infiltrates semi-quantitative analysis, whole sections were examined.

### In situ hybridization and immunohistochemistry

SIV gag *in situ* hybridization combined with immunohistochemistry for cell markers was performed as previously described [44], using a 743 bp SIVmac251 gag cDNA fragment (Genbank accession number M19499, nucleotides 1386-2129) to generate 35S-UTP-labeled riboprobes. The specificity of the hybridization signal was systematically checked by hybridizing sense probes on parallel sections and anti-sense probes on uninfected genital tissues. SIV positive cells co-labelled or not with cell markers were counted in a minimum of 30 adjacent sections/experiment, in three independent experiments. The total surface area counted was determined using the Cast Grid software (Olympus, France).

### Cytokine mRNAs quantification

TaqMan quantitative real-time PCR assays were performed on 100 ng (cytokine) or 40 ng (18 s) of equivalent RNA from 2 independent tissue fragments run in duplicate with the ABI7500 using commercially available master mix and following human target probes (Applied Biosystems): Hs00174097_m1 (IL1β), HsOO174086_m1 (IL10), Hs00998133_m1 (TGFβ), Hs99999901 _s1 (18s). Primers and probes for simian IFNγ and TNFα were described elsewhere [45]. The Ct values of each gene were calculated with the ABI Sequence Detection System 1.9 program and normalized to the level of 18s RNA. The absolute gene expression level was calculated by the standard curve method using plasmid standards encoding for simian IFNγ, TNFα, IL1β, IL10 and TGFβ (kindly provided by Dr Villinger, Emory University School of Medicine, Atlanta, USA) or PCR product (18 s). Results were expressed as copy numbers of the mRNA of interest per copy of 18s RNA.

### Hormone assays

The testosterone and LH assays were conducted in the Yerkes National Primate Research Center (Emory University, Atlanta, USA) on macaques' serum samples collected prior and after infection. Serum levels of testosterone were measured by RIA (Diagnostic Systems Laboratories, Webster, TX). The sensitivity of the assay was 0.05 ng/ml; the intra- and interassay coefficients of variations were 6.3% and 5.95% at 0.68 ng/ml and 4.14% at 5.67 ng/ml, respectively. Serum levels of LH were measured by a mouse Leydig cells bioassay, as previously described [46]. The sensitivity of the assay was 0.2 ng/ml; the intra- and interassay coefficients of variations were 5.44% and 18.4%, respectively. Samples were assayed in duplicates and results expressed as the median +/− quartiles for each time point.

### Statistical analyses

The non-parametric unpaired Kruskal-Wallis test was used to assess differences according to infection status/blood viremia (SIV DNA detection, cytokine quantification) whilst the non-parametric paired Wilcoxon test was used to assess differences within one animal group (variation in SIV DNA detection and cell quantification amongst organs, hormones level variations during time course). Coefficients of correlation (r) between blood viremia and frequency of detection of SIV in male genital organs (nested PCR for SIV DNA) were calculated using the Spearman rank test. All analyses were performed using the statistical software R.

## Results

### Identification and quantification of SIV/HIV target cells

**Figure 1:**
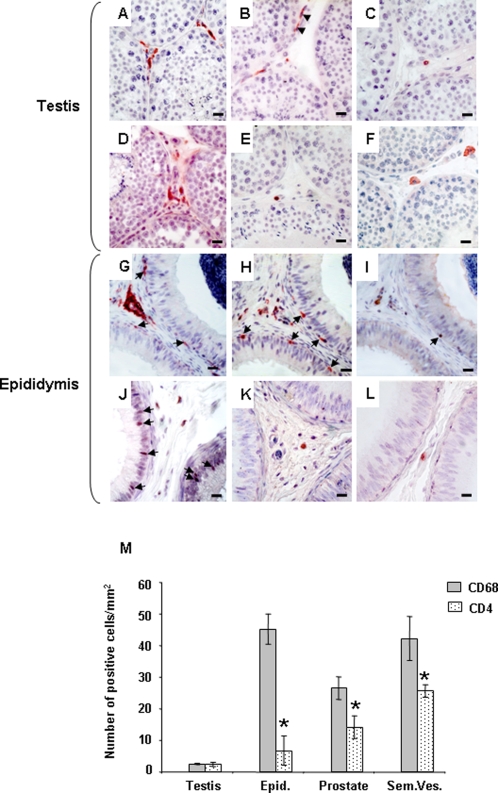
Localization and quantification of SIV/HIV target cells in the male genital tract. Testis (A–F) and epididymis (G–L) immunolocalization of HLA-DR (A, G), CD68 (B, H), CD3 (C, I), CD4 (D, J), CCR5 (E, K) and CXCR4 (F, L) positive cells in uninfected macaques. Arrows show immunopositive cells in contact with the epithelium of the epididymis. Note the presence of testicular macrophages within the peritubular wall bordering the seminiferous tubules of the testis (Figure 1H, arrow heads).Scale bars = 20 µm. (M): Quantification of HIV potential target cells (CD68+ and CD4+ stained positive cells) in the male reproductive organs of uninfected macaques. Stars indicate statistical difference between the number of CD68+ cells and CD4+ cells within an organ (Wilcoxon signed rank test, p<0.05;). The number of CD68+ cells was significantly lower in the testis when compared with the other MGT organs (Wilcoxon signed rank test, p<0.05, not shown on the graph). The number of CD4+ cells in the testis was significantly lower than in the prostate and seminal vesicles (Wilcoxon signed rank test, p<0.05, not shown on the graph).

Throughout the MGT of uninfected macaques, immune cell subsets and localization were similar to previous observations in humans [47], [48]: HLA-DR+ cells, CD3+ T lymphocytes, CD68+ macrophages and CD4+ cells were found within the testicular interstitial tissue (Figure 1A–C), the stroma of the epididymis (Figure 1G–I) and accessory glands (data not shown) and inserted within the epididymal epithelial cells (Figure 1G–I). Similar localization was observed for CCR5+ and CXCR4+ cells (Figure 1D–F, J–L). Quantification of potential HIV target cells (i.e. macrophages and CD4+ T lymphocytes) revealed that the testis displayed significantly lower number of macrophages than the other organs (on average over 16 fold less than in epididymis and seminal vesicles and 10 fold less than in prostate) (Figure 1M). CD4+ cells were also lower in the testis than in the seminal vesicles (on average 10 fold less), the prostate (6 fold less) and epididymis (3 fold less), although the difference was not statistically significant for the latter (Figure 1M). In all organs but testis, macrophages predominated over CD4+ T cells (Figure 1M).

### Characteristics of infected animals

**Figure 2:**
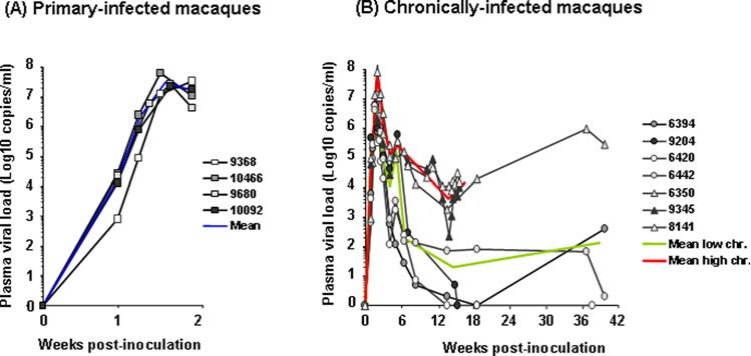
Blood PVLs from time of intravenous SIVmac251 inoculation to necropsy. (A) Four animals were sacrificed at 14 days p.i.; (B) Seven animals, sacrificed between 15 to 37 weeks p.i., were distinguished into one group of 3 “high chronic animals” (triangles) and one group of 4 “low chronics” (circles), based on PVL at necropsy. Mean viral loads are represented by a blue line for the primary-infected, a red line for the chronically-infected animals with high PVL and a green line for the chronically infected animals with low PVL.

Four macaques were euthanized at peak blood viremia (Figure 2A) [named below “primary-infected macaques”], and seven were euthanized during the chronic asymptomatic stage (Figure 2B). Amongst the latter, two groups were distinguished (Figure 2B): -four macaques displayed a blood plasma viral load (PVL)<4 Log six weeks post-infection (p.i.) (a time point at which viremia level is highly predictive of the outcome of the infection [49]). Two of these four animals had completely controlled viremia at the time of sacrifice (Figure 2B, animal 6420 & 9204), whilst the other two (6442 & 6394) presented a PVL<3 Log. These four animals were called “low chronics” in subsequent analysis; -three animals, classified as “high chronics”, displayed a PVL>5 Log by six weeks post-infection and still displayed PVL>4 Log at the time of sacrifice. Of note, no persistent CD4+ cell depletion was observed in any of the chronically-infected macaques tested (refer to Figure S1 for CD4+T cell counts of primary and chronically-infected animals).

### Detection of viral DNA

**Figure 3:**
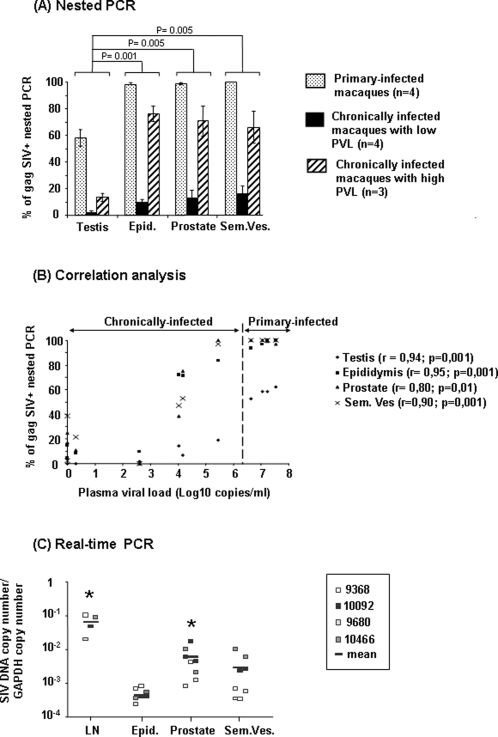
SIV DNA detection and quantification in the male genital organs. (A) Frequency of detection of SIV DNA in the testis, epididymis (Epid.), prostate and seminal vesicle (Sem. Ves.) of SIV-infected macaques, using nested SIV gag PCR. Each bar represents the mean +/− SEM of gag+ PCR for each organ within a group of animals. Statistical difference (Kruskal Wallis test, p<0.05) was found between the MGT of primary-infected, high chronic and low chronic animals (not shown on the graph); p values shown on the graph indicate statistical difference between the organs, according to Wilcoxon signed rank test. (B) Frequencies of detection of SIV gag DNA in genital organs of primary and chronically-infected animals were tested for association with blood viremia by Spearman rank test. The different organs are depicted by different symbols. Positive correlation was found for all male genital tract organs levels of infection and blood viral load. (C) SIV DNA viral load in mesenteric lymph nodes (LN), epididymides, prostate and seminal vesicles of primary SIV-infected macaques, in quantitative real time PCR. Mean of 4 animals is represented by black bars. Squares with the same pattern show viral load for 2 independent fragments of the same organ. Stars indicate statistical difference between the epididymis and the other organs (Wilcoxon signed rank test, p<0.05).

Nested PCR detected SIV-DNA in the testis, epididymis and accessory glands of all acutely-infected animals (Figure 3A). During this phase, SIV detection rate in the MGT was significantly higher than during chronic phase. Furthermore, MGT infection was significantly reduced in low chronic animals when compared to high chronics (Figure 3A). A positive correlation was found between the frequency of detection of SIV in the MGT and blood viremia (Figure 3B). Throughout the infection, the testis displayed the lowest rate of infection amongst the organs tested, a finding confirmed in primary-infected animals by measurement of reproductive tissues viral load (VL) in real time PCR: prostate and seminal vesicles VL were on average 1 Log higher than epididymis mean VL (Figure 3C), whilst testis VL was consistently below the quantification threshold of the real time PCR. Of note, semen-producing organs VLs were at least 1 Log lower than mesenteric lymph node VL.

### SIV localization

**Figure 4:**
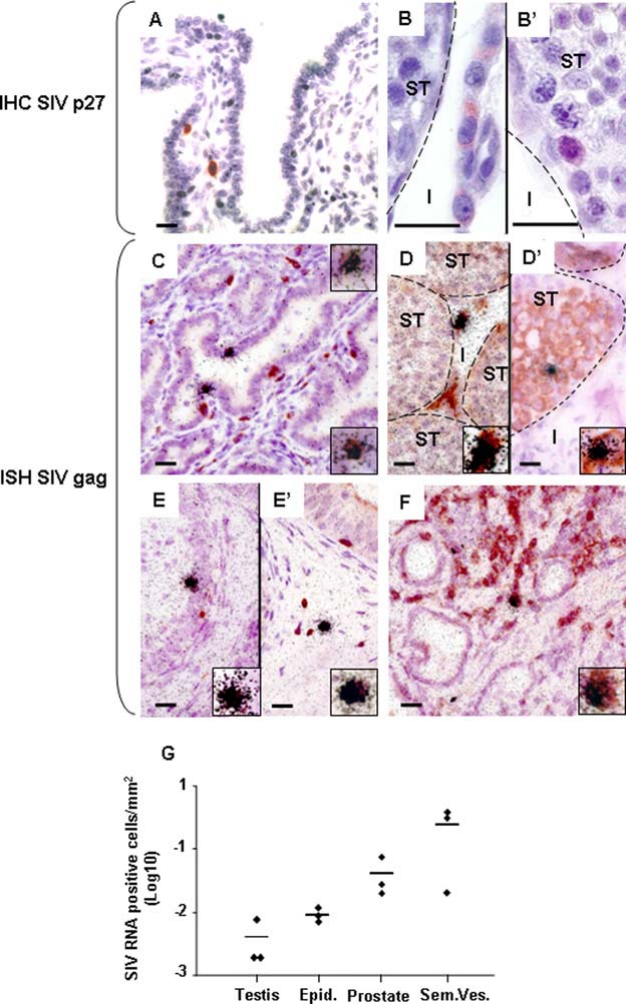
SIV localization within the male genital tract. Detection of SIV positive cells in the seminal vesicles (A, C), testes (B–B', D–D'), epididymides (E, E') and prostate (F) of primary-infected macaques using immunohistochemistry for SIVp27 (A, B) and in situ hybridization (ISH) for SIV gag RNA (C–F). The phenotype of SIV positive cells was determined using ISH for SIV gag RNA (visualized as black silver grains) combined with immunostaining for cell markers (visualized as brown staining): combined ISH for viral RNA and immunostaining for CD3 revealed black silver grains clustered over brown cells in the seminal vesicles (C), prostate (F), and epididymis (E'), indicating infection of CD3+T lymphocytes. Co-labelling of SIV RNA+ cells with the myeloid cell marker CD68 was also observed, as shown here for the epididymis (E). In the testis, SIV RNA was detected within the interstitium in HLA-DR+ cells (D) and within the seminiferous tubules in VASA+ germ cells (D'). Inserts show enlargement of SIV RNA positive cells co-stained for cell markers. I: testicular interstitium; ST: seminiferous tubules. Scale bars = 20 µm. (G) SIV RNA+ cells were counted in a minimum of 30 tissue sections/experiment in 3 independent experiments on a primary-infected macaque MGT. Results show the mean positive cell number/tissue area +/− SEM.

SIV p27 positive cells were found within the stroma and close to the secretory epithelium of the seminal vesicles (Figure 4A), prostate and epididymides (data not shown) of primary-infected animals. Positive staining was observed in the interstitium and seminiferous tubules of the testis (Figure 4B, B'). *In situ* hybridization for SIV gag RNA also revealed positive cells in the stroma and, occasionally, within the epithelium of the seminal vesicles (Figure 4C), epididymides (Figure 4E, E') and prostate (Figure 4F) of primary-infected animals. Within the testis, SIV RNA+cells were observed in the interstitium (Figure 4D), occasionally bordering the seminiferous tubules (data not shown). In all tissues, these infected cells were mainly T lymphocytes (60–97% of SIV RNA+ cells co-labeled with CD3, the highest proportion being found in the prostate and seminal vesicles) (Figure 4C, E', F), and some macrophages (0–25% of cells co-labeled with CD68, the highest rate being consistently found in the epididymis) (Figure 4E). Interestingly, in the testis SIV gag positive cells that never co-localized with either HLA-DR, CD3 or CD68 were occasionally observed within the seminiferous tubules (Figure 4D') (on average 1 positive cell for 300 seminiferous tubules). These positive cells systematically co-localized with VASA, a specific germ cell marker [42]. Their distribution and localization, from the base to the middle of the tubules, suggested an association of SIV with pre-meiotic and meiotic germ cells. In chronically infected macaques, the same pattern of SIV localization was observed, but fewer cells were affected. Importantly, however, a few SIV+ cells were detected within the MGT of an animal with undetectable blood viremia (data not shown). A quantitative measurement of the number of SIV+ cells per tissue surface in a primary-infected animal evidenced that seminal vesicles and prostate displayed a higher number of positive cells when compared to the epididymides and testes (Figure 4G).

### Viral populations in the MGT

**Figure 5:**
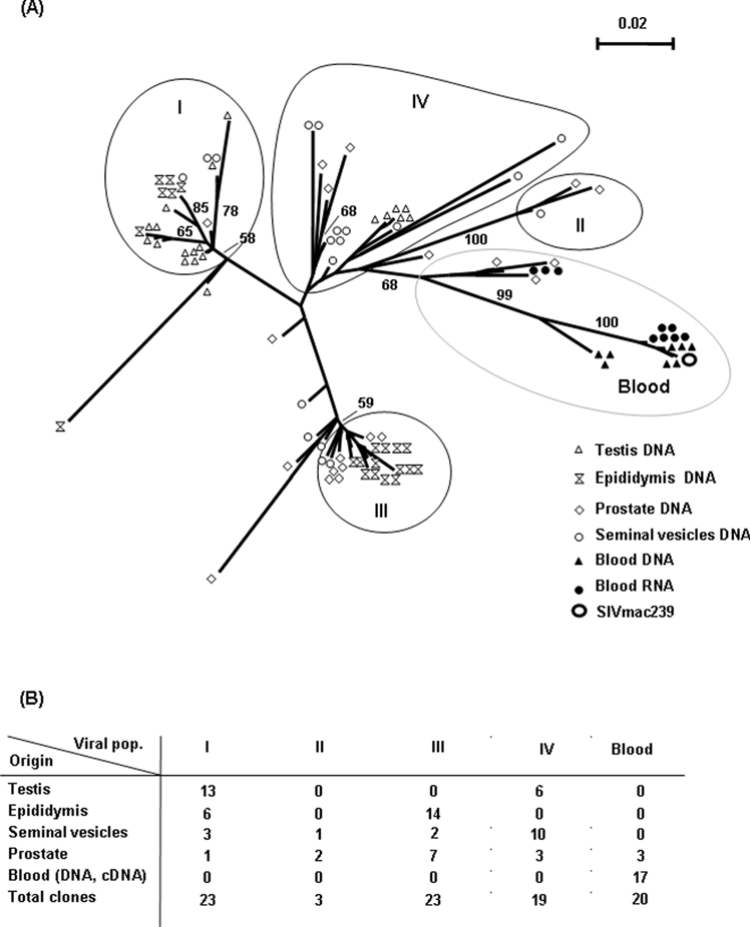
Viral populations in the MGT. (A) Phylogenetic analyses of V1-V2 sequences from quasi-species obtained from reproductive tissues (white symbols) and blood (black symbols) of a high chronic macaque at necropsy. Trees were built with PAUP* version 4b10. Major significant phylogenetic clusters in the MGT are rounded in black and numbered I to IV. The numbers near nodes indicate the percentage of bootstrap replicate (100 resamplings). The scale refers to the distance between sequences. (B) Tissue origins of the clones present in the different viral populations.

The genotype of the virus strains present in the reproductive organs (obtained from different PCR rounds and pooled) was compared with those isolated from PBMCs and blood plasma in one high viral load chronically-infected macaque (Figure 5). We evidenced four main clusters of clones in the MGT, which were linked, but statistically distant from, the main viral population present in the blood, as indicated by the high bootstrap value 99, 100. However, a few sequences from the prostate were close to RNA sequences from blood, suggesting a continuous re-seeding of the resident sequences in MGT. Interestingly, only a partial segregation could be seen between the different compartments of the reproductive tissues: the neighbour-joined tree showed highly heterogeneous viral populations that could be separated into four major clusters closely related altogether (Figure 5A). However, we observed a relative enrichment of some tissue specific clones in the different populations (Figure 5B). Thus epididymis sequences are found mainly in cluster III and from seminal vesicle in population IV. Similar observations were done in a low viral load chronically infected animal despite a lower number of available sequences (Figure S2).

### Local immune responses in the MGT

**Figure 6:**
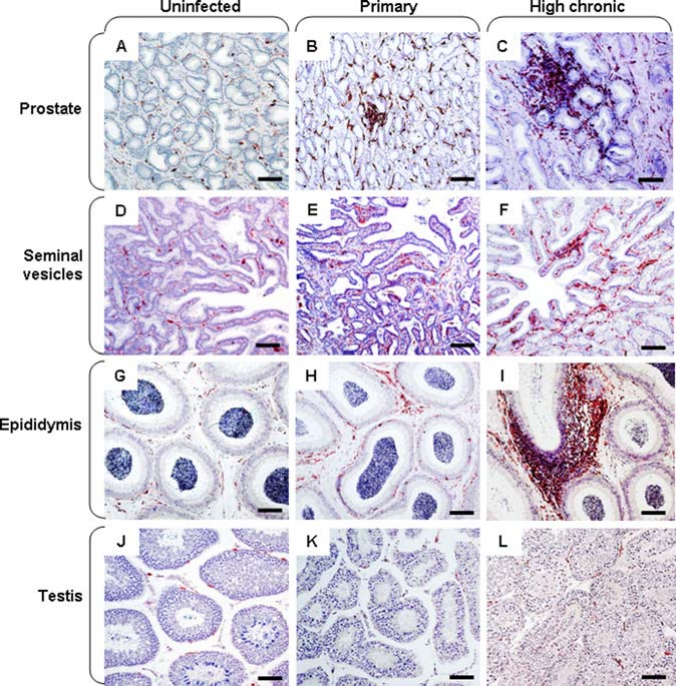
Immune activation in the male genital organs. Immunohistochemical detection of HLA-DR+ cells in the prostate (A–C), seminal vesicle (D–F), epididymis (G–I) and testis (J–L) of uninfected (A, D, G, J), primary-infected (B, E, H, K) or high chronic macaques (C, F, I, L). Scale bars = 100 µm.

**Figure 7:**
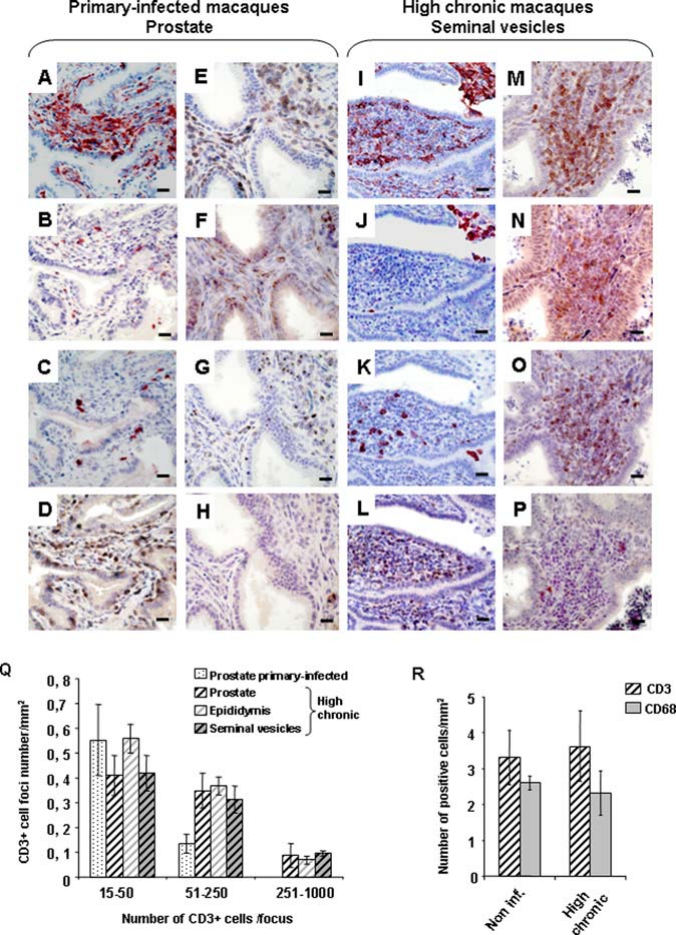
Characterization and semi-quantitative analysis of immune response in the infected reproductive organs. (A–P) Immunohistochemical characterization of immune cells present in the prostate of primary-infected macaques (A–H) and in the seminal vesicle of chronically infected macaques with high blood viremia (I–P): serial tissue sections (each column represents 4 serial sections) were stained with anti-HLA-DR (A, I), anti-CD68 (B, J), anti-CD20 (C, K), anti-CD3 (D, E, L, M), anti-CD4 (F, N), anti-TIA-1 (G,O) or anti-Pen5 (natural killer marker) antibodies (H,P). Note the presence of HLA-DR+ and CD68+ cells in the seminal vesicle lumen (I, J). Scale bars = 20 µm. (Q) Semi-quantitative analysis of CD3+ cell foci in the male genital organs of infected macaques. The number of CD3+ cells in each focus was determined using the Cast software. The number of foci in each category (15–50 cells, 51–250 cells, 251–1000 cells) was counted on whole sections of epididymis, seminal vesicles and prostate from chronically-infected macaques with high PVL, and prostate from primary infected macaques. (R) Quantitative analysis of CD68+ and CD3+ stained positive cells in the testes of non-infected macaques (non inf.) and macaques chronically infected with SIV and displaying high PVL (high chronic).

The morphology of the seminal vesicles, epididymides and testes was similar in acutely-infected animals (Figure 6E, H, K) when compared to uninfected ones (Figure 6D, G, J). However, HLA-DR+ cell foci were observed within the prostate during the acute phase of the infection (Figure 6B versus Figure 6A). In low chronic animals, cell foci were never observed (data not shown). In contrast, in high chronic animals, outsized HLA-DR+ cell infiltrates were observed in the stroma of the prostate (Figure 6C), seminal vesicles (Figure 6F) and epididymides (Figure 6I), but were never found within the testis (Figure 6L). During both the primary and chronic stage of the disease, the cellular infiltrates were mainly composed of CD3+ T lymphocytes and comprised a mix of CD4+ T helper and TIA-1+ cytotoxic cells (Figure 7E, F, G and M, N, O) whilst only very few cells labelled with the NK marker Pen5 (Figure 7H and P); only a few CD20+ B lymphocytes (Figure 7C and K) and CD68+ myeloid cells (Figure 7B and J) were present. Semi-quantitative analysis indicated that there were no marked differences between the sizes and number of CD3+ T cell foci amongst the epididymis and accessory glands of chronically-infected macaques with high PVL. Although the prostate of primary-infected animals displayed similar numbers of small size CD3+ infiltrates (15–50 cells) than the prostate of high chronic macaques, medium size foci (51–250 cells) were 2.7 fold less numerous and large size foci (251–1000 cells) were never encountered (Figure 7Q).

Testes of chronically infected animals with high blood viremia displayed similar number of CD3+ T cells and CD68+ macrophages than the uninfected ones (Figure 7R).

**Figure 8:**
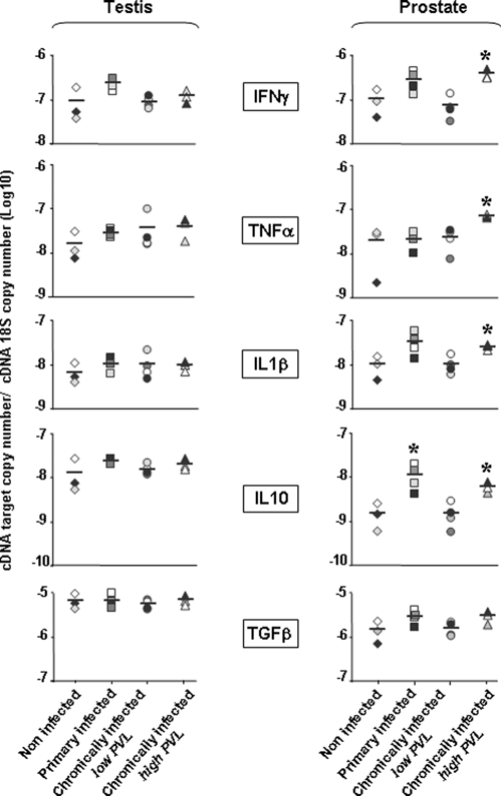
Cytokine mRNAs expression. Pro-inflammatory and immunosuppressive cytokines transcripts expression in testis and prostate tissues, as measured by quantitative real time RT-PCR. Each point represents the mean cytokine copy number of 2 independent fragments from one animal. Stars indicate statistical difference between non-infected and infected-macaques (Kruskal-Wallis test, p<0.05).

Within the testis, the levels of expression of the immunosuppressive cytokines IL-10 and TGFβ and pro-inflammatory cytokines IFNγ, IL-1β, TNFα transcripts was not significantly changed following infection (Figure 8).

Of note, IL-10 and TGFβ mRNAs levels in both the uninfected and infected testis were about 10 fold that of uninfected prostate, in concordance with the previously reported immuno-suppressed status of this organ [48]. In the prostate of primary-infected macaques, a significant increase in IL-10 transcript expression was detected when compared to uninfected animals, while the expression of other cytokines was not significantly modified (Figure 8). In contrast, in addition to IL-10, IFNγ, TNFα and IL-1β mRNAs were significantly enhanced in the prostate of high chronic macaques (Figure 8). In the prostate of low chronic animals, cytokine mRNAs expression was similar to that of uninfected animals, suggesting that the absence of cell infiltrates was due to lack of local immune activation rather than to an enhanced immunosuppressive response (Figure 8).

### Impact of the infection on the exocrine and endocrine testicular functions

**Figure 9:**
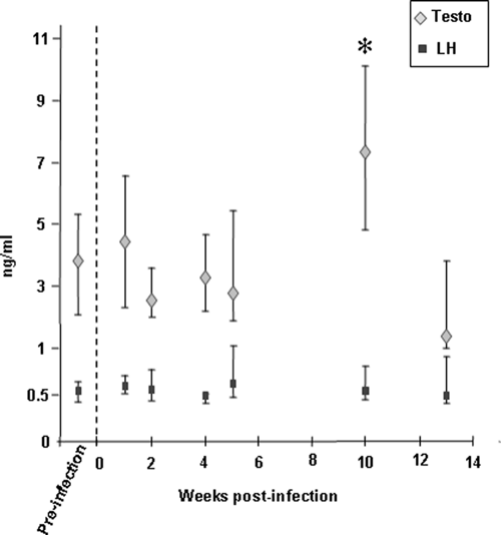
Testosterone and LH levels measured in macaques' serum. Results are expressed as the median value +/− Q3 and Q1 quartiles. Pre-infection testosterone and LH values each represents the pool of three measures performed on twelve macaques at different time points prior to infection. For each time point p.i., 12 animals were tested. * indicates statistical difference to the pre-infection level (Wilcoxon test, p<0,05).

Normal spermatogenesis was observed in the testis of all infected animals (Figure 6K, L) and packs of spermatozoa were found within the epididymis lumen (Figure 6H, I) indicating that the infection did not impair sperm production. Sperm quality was not assessed. Interestingly, an increase in testosteronemia was observed 10 weeks p.i., while luteinizing hormone (LH) level remained unchanged (Figure 9).

## Discussion

Understanding the spacio-temporal colonization of the MGT by HIV is crucial in any attempt to prevent its transmission and to improve the antiretroviral therapies. The few studies that addressed this question have focused mainly on the late stage of the disease (reviewed in [50]). Using a macaque model, this work provides the first extensive description of SIV interactions with a number of organs crucially involved in the production of semen, both during the acute and chronic asymptomatic phases of the infection.

We demonstrate the presence of SIV in the testes, epididymides, prostate and seminal vesicles of acutely and chronically-infected macaques, using different but complimentary techniques. Similar early viral dissemination had been reported for several other lymphoid and non-lymphoid organs, independent of the route of infection [51]–[53]. For the first time, we establish a positive correlation between the frequency of detection of SIV in the reproductive organs and plasma viremia throughout the infection. Of note is that such a correlation had been described for semen from men and macaques during the acute [26], [54] and chronic stage of the infection [4], [55]–[57], as well as for other tissues [58]. Virus colonizes the male genital tract during the primary infection, at a time when blood viral load is at its peak and thus likely to favour diffusion of free viral particles and infected leukocytes amongst the organs.

Markedly different levels of infection were found amongst the semen-producing organs. The prostate and seminal vesicles appeared the most highly infected organs, followed by the epididymis, while the testis consistently displayed the lowest infection rate. In rhesus macaques with AIDS, the testis was similarly found to be the least infected organ within the MGT [25]. This low level of infection probably results from the overall smaller number of potential target cells (i.e. macrophages and CD4+ cells) that we evidenced in the uninfected testes when compared to the other MGT organs, as previously described in humans [48]. In addition, the testis specific environment is likely to reduce immune cell infectivity *in situ* and infected cell migration in this organ. Thus androgens and testicular immunosuppressive factors are known to inhibit inflammation and T-cell activity in the testis [59]–[63]. Indeed, immune cell infiltrates were never encountered in the infected testis.

In our study, T lymphocytes represented the main infected cell population throughout the MGT while macrophages were predominant over CD4+ T cells in the epididymis and accessory glands prior to infection. This may reflect the migration of blood-borne infected lymphocytes into these tissues and/or the preferential infection of this cell type rather than resident macrophages. In contrast, earlier studies on the MGT of men and rhesus macaques with AIDS described macrophages as the most infected cell types throughout the MGT [23], [25]. In AIDS rhesus macaques, epididymis was the most infected organ [25], as opposed to the prostate and seminal vesicles in our study. These differences could reflect CD4+ T lymphocytes depletion at the late stage of the disease. Thus, the persistence of infected macrophages in the MGT tissues whilst CD4+T cells are depleted may induce modifications in tissue viral load. In asymptomatic SIV and SHIV-infected pigtail macaques, both infected T lymphocytes and macrophages were found in the testis and epididymis [64]. Importantly, it has been demonstrated that experimental infection of cynomolgus macaques from Mauritius with SIVmac251, as used in this study, leads to virological and immunological evolution profiles that more closely mimics HIV infection in humans than the model of SIV-infected rhesus macaques of Indian origin [33], [65]–[67]. Indeed, infection in rhesus macaques of Indian origin is characterized by profiles of high viral load and rapid decrease of CD4+ T cell counts associated with atypical anti-SIV immune response that may not be relevant to the human situation [67].

Inflammatory lesions have previously been evidenced at all levels of the reproductive tract of men and macaques with AIDS [23], [25]. In our study, acutely-infected animals did not display any inflammatory foci in the epididymis and seminal vesicles and only small/medium size cell foci were detected in the prostate. Pro-inflammatory cytokine IL-1β, IFNγ and TNFα mRNAs levels were not significantly modified in the prostate of these animals compared with uninfected ones, while the immunosuppressive cytokine IL-10 mRNA expression was enhanced. This differs from the situation described in PBMCs and lymph nodes of acutely-infected cynomolgus macaques [68], [69] and humans [70]–[72] where increased expression of both pro-inflammatory and immunosuppressive cytokine mRNAs was reported. Of note is that pro-inflammatory cytokines expression in tissues has been shown to positively correlate viral RNA level [73]. Thus, the low level of infection of MGT organs may not be sufficient to trigger their expression during the acute stage. In contrast, in the prostate of chronically-infected macaques with high PVL, both IL-10 and pro-inflammatory cytokines mRNAs were found to be elevated. This was associated with enlarged CD3+ cell foci composed of activated helper and cytotoxic T lymphocytes and may reflect a high level of activation of the immune system in these animals. The persistent recruitment of activated T cells to male genital organs is likely to increase the tissue viral load by providing a source of target cells for the virus. Although it cannot be ascertain, the enhanced expression of IL-10, IFNγ, TNFα and IL-1β transcripts in the prostate may reflect protein expression as all these cytokines are known to be up-regulated in immune cells during the course of HIV and SIV infection [74], [75]. Moreover, in HIV negative men with chronic inflammation of the prostate, elevated levels of IL-10 and inflammatory cytokines are found in the seminal plasma [76]–[78], indicating protein production. In contrast to macaques with relatively high level of MGT infection, neither immune infiltrates nor changes in cytokine expression levels were observed in the organs of the animals displaying low level MGT infection. This strongly suggests that local immune activation represents an important determinant of the tissue viral load in chronic animals.

No immune response was observed in the testis throughout the infection, in contrast to the other MGT organs. Although this could be due to the very low level of testicular infection, it is likely that the immuno-protected status of the testis plays an important role [48]. Amongst the various mechanisms that attenuate immune responses in this organ [48], [79]–[84], elevated levels of the immunosuppressive TGFβ together with diminished levels of pro-inflammatory cytokines expression have been reported following bacterial exposure [84]. This is consistent with our results in the SIV-infected macaques testis.

For a number of years, the question as to whether blood and MGT represent distinct viral compartments has been debated (reviewed in [50], [85]). In the subject study, the presence of productively-infected cells in the MGT of an animal with undetectable blood viremia, further reinforces the previous indirect evidence indicating viral compartmentalization within the MGT [50], [85]. Other evidence that viral compartmentalization exists was our finding that MGT and blood viral populations were linked but clustered distinctly. This indicates that local virus production and blood re-seeding occur and explains the previous findings of both different [1]–[13] and identical [1], [86] HIV-1 variants in semen and in blood. Interestingly, some of the male genital tract specific viral populations were shared amongst the reproductive organs, suggesting free virus drainage and/or infected cell migration at this level. Similar intermingling had been reported in human prostate and testis from HIV-1 infected individuals [87]. This could result from viral particles and infected cells circulation within the MGT, due to the inter-vascular connections which exist in between the reproductive organs [88]. It may also be facilitated by the transit of secretions and cells along the excretory ducts during ejaculation, as well as by retrograde contamination of these organs [89]. Independent evolution of viral variants in the MGT may induce the formation of variants that harbour the ability to escape the effects of immune system and drugs, vary tropism and pathogenicity. In presence of sub-optimal drug penetration, the MGT could participate in seeding the systemic compartment.

The detection of infected immune cells adhering to the epididymides and accessory glands epithelium of the SIV-infected macaques suggests that virus particles and infected cells are susceptible to be released into the seminal fluid and contribute to its viral load, as described in other tissues (reviewed in [90]). Since the seminal vesicles and prostate represent the two most infected organs and their respective secretions constitute 60% and 30% of the seminal fluid [91], they are likely to be the source of most viral particles in the semen. In favor of this hypothesis, prostate massage has been shown to significantly increase HIV RNA shedding in seminal plasma [92]. The lower level of infection of the testes and epididymides detected here strongly suggests that these organs are lower contributors to the viral load in semen. This is compatible with the fact that vasectomy has little effect on the level of seminal HIV RNA [93], [94]. It is believed that the epididymis represents the main source of lymphocytes and macrophages in semen [95]. Indeed, our results show that it is in this organ that infected T lymphocytes and macrophages are the most frequently encountered within the secretory epithelium. Our findings that T lymphocytes represent the predominant infected cell type in the MGT also correlates with the fact that infected T cells are the main infected cell population in semen [96].

Whether or not testicular germ cells can be contaminated in humans has been controversial (pro: [22], [24], [97], [98]; anti: [23], [87], [99]). We demonstrate here that occasional detection of SIV RNA and antigens associated with isolated germ cells within the seminiferous tubules of acutely and chronically-infected macaques can occur. This is in agreement with another recent finding, in post-acute asymptomatic SIV and SHIV-infected pigtail macaques [64], but contrast with an earlier study on moribund SIV-infected rhesus macaques [25]. This latter discrepancy could be explained by differences in technique sensitivities, sizes of sampling, testicular morphology according to disease stage or species. How HIV/SIV binds and/or infects germ cells remains unknown. Using immunohistochemistry, we did not find any expression of CD4, CXCR4 and CCR5 in the seminiferous tubules. However, germ cells may express low levels of one or several of those receptors that would not be detected using this technique. The alternative HIV receptor GalCer has previously been detected on germ cells [100] and could allow HIV binding and/or infection of these cells [101]–[103]. The presence of infected immune cells within the peritubular wall could also contribute to virus transmission to germ cells.

The present study establishes that normal spermatogenesis occurs in the acute and chronically SIV-infected macaques. A finding similar was reported in asymptomatic men in whom infected germ cell have been detected [98]. Furthermore we observed a transient increase in testosteronemia during the post-acute stage in macaques. This is likely to reflect a direct effect of the infection on Leydig cell steroidogenesis as no change in LH serum levels was observed. However, we did not find infection of Leydig cells in our SIV-infected macaques, in agreement with our previous work on HIV/SIV infection of human testis [43], [104]. As no modification in the production of cytokines was observed in the testis of infected macaques, the observed testosterone increase is likely to result from direct interactions of infected macrophages/lymphocytes with Leydig cells [105], or from secretion of unidentified factors [106]. The effect of the infection on sperm quality could not be assessed in this study as semen was not available. Of note is that in HIV-1+ men, semen parameters can be altered [50], [107]–[109]. However it is not clear whether this results from the infection itself or from the antiretroviral treatments. In men and macaques suffering from AIDS, various stages of germ cell degeneration were reported (reviewed in [85]). This probably results from the decreased testosterone levels often observed in these patients (reviewed in [85]).

In conclusion, the present study reveals that SIV infection of the macaque MGT is an early event and that semen-producing organs display differential infection levels and immune responses. Although there may be differences between humans and macaques, these results strongly suggest that in the absence of treatment, several male genital organs may be involved in HIV shedding in semen throughout the infection. Analyses of split ejaculates from infected macaques would be useful to further trace the source of infected cells in semen back to specific organs in the MGT. Our data pave the way for further experiments aimed at establishing whether one or several of these organs constitute a viral reservoir(s) that could lead to the persistent seminal HIV shedding observed in men under antiretroviral therapy [3], [14]–[18], such as the analysis of SIV infection of male genital organs and semen of macaques under HAART. Identifying the nature of the viral sanctuaries within the MGT is of crucial importance for the design of new antiviral therapies.

## Supporting Information

Figure S1 CD4+ T cell counts in primary-infected macaques (A) and chronically-infected macaques with high plasma viral load (PVL) (B) or with low PVL (C). CD4+ cells were measured in the blood of macaques at several time points prior (Pre-inf.) and post-infection (Post-inf.) and at the time of euthanasia (except for animals 6420 & 6442, data not available at the time of euthanasia). The different animals are depicted by different symbols.
(0.07 MB TIF)

Figure S2 (A) Phylogenetic analyses of V1-V2 sequences from quasi-species obtained from testis, prostate and seminal vesicle tissues (white symbols) and blood (black symbols) of a low viral load chronically infected macaque at necropsy. No viral DNA was isolated from the epididymis tissue of this animal. Trees were built with PAUP* version 4b10. Major significant phylogenetic clusters in the MGT are rounded in black and numbered I to IV. The numbers near nodes indicate the percentage of bootstrap replicate (100 resamplings). The scale refers to the distance between sequences. (B) Tissue origins of the clones present in the different viral populations.
(0.08 MB TIF)
