# Supplementary figures and images for: Infection of Semen-Producing Organs by SIV during the Acute and Chronic Stages of the Disease
Source: PLoS One. 2008 Mar 12;3(3):e1792. doi: 10.1371/journal.pone.0001792 (PMC2268241; doi:10.1371/journal.pone.0001792)

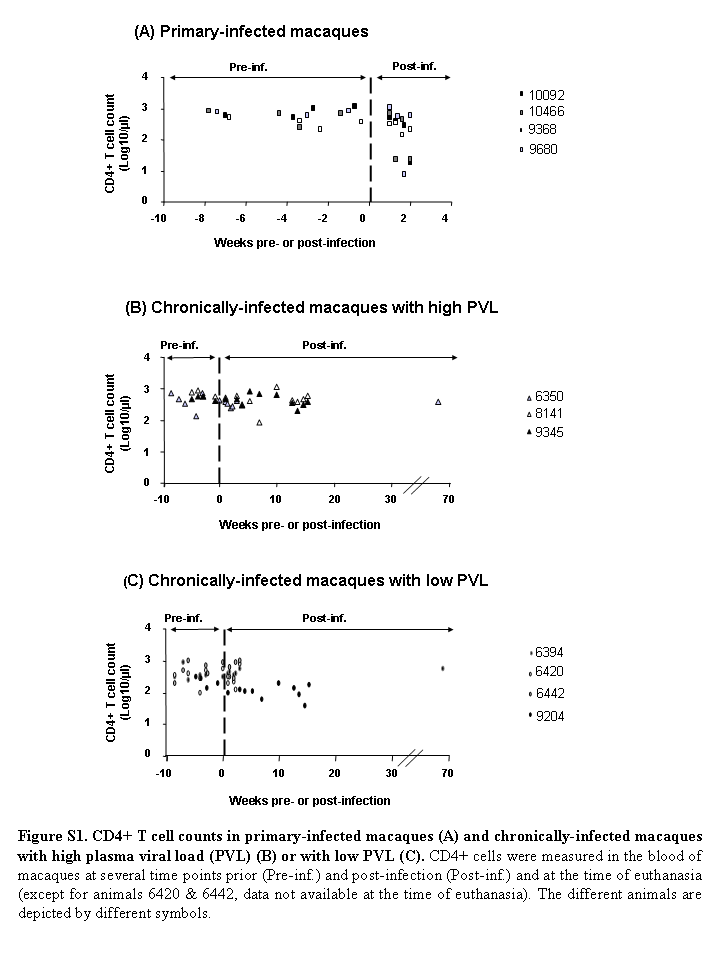

Supplement: Figure S1 — CD4+ T cell counts in primary-infected macaques (A) and chronically-infected macaques with high plasma viral load (PVL) (B) or with low PVL (C). CD4+ cells were measured in the blood of macaques at several time points prior (Pre-inf.) and post-infection (Post-inf.) and at the time of euthanasia (except for animals 6420 & 6442, data not available at the time of euthanasia). The different animals are depicted by different symbols. (0.07 MB TIF) [file pone.0001792.s001.tif]

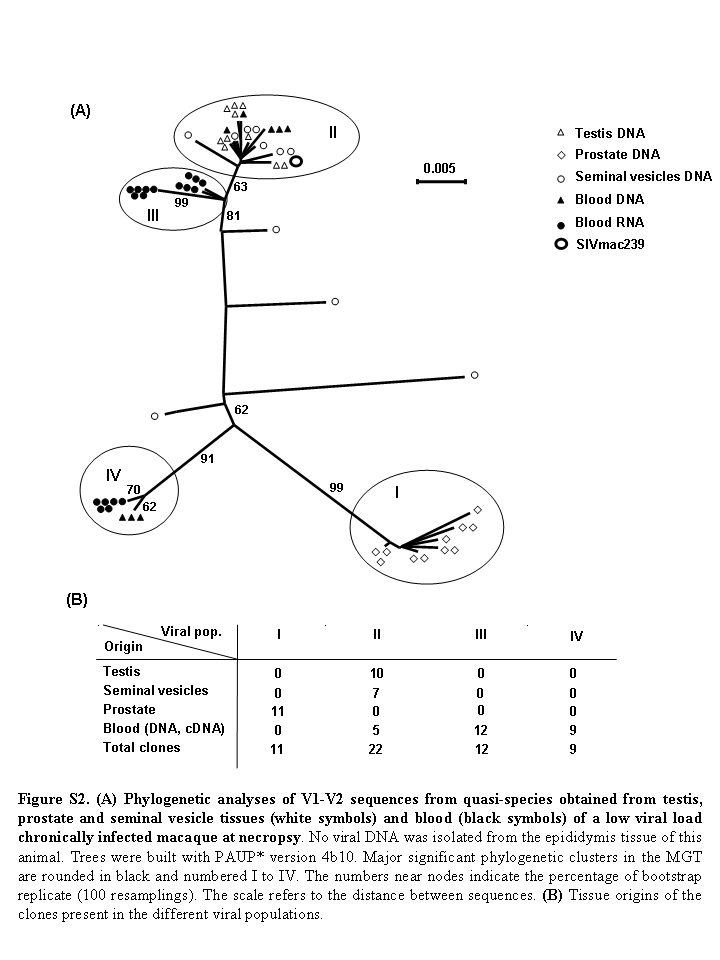

Supplement: Figure S2 — (A) Phylogenetic analyses of V1-V2 sequences from quasi-species obtained from testis, prostate and seminal vesicle tissues (white symbols) and blood (black symbols) of a low viral load chronically infected macaque at necropsy. No viral DNA was isolated from the epididymis tissue of this animal. Trees were built with PAUP* version 4b10. Major significant phylogenetic clusters in the MGT are rounded in black and numbered I to IV. The numbers near nodes indicate the percentage of bootstrap replicate (100 resamplings). The scale refers to the distance between sequences. (B) Tissue origins of the clones present in the different viral populations. (0.08 MB TIF) [file pone.0001792.s002.tif]
